# A study investigating the treatment of equine squamous gastric disease with long‐acting injectable or oral omeprazole

**DOI:** 10.1002/vms3.220

**Published:** 2020-01-16

**Authors:** Sarah Gough, Gayle Hallowell, David Rendle

**Affiliations:** ^1^ Rainbow Equine Hospital Malton North Yorkshire United Kingdom; ^2^ School of Veterinary Medicine and Science University of Nottingham Nottingham United Kingdom

**Keywords:** gastrointestinal, horse, proton pump inhibitor, stomach

## Abstract

**Background:**

Equine squamous gastric disease (ESGD) is a highly prevalent disease in horses, particularly in elite athletes. Some horses respond slowly, or fail to respond, to the licensed treatment, oral omeprazole (ORLO).

**Objectives:**

To compare rates of ESGD healing and improvement between ORLO and a long‐acting injectable omeprazole preparation (LAIO).

**Study design:**

Retrospective clinical study.

**Methods:**

The case records and gastroscopy images of horses presenting to Rainbow Equine Hospital over a 12‐month period were reviewed, with images being reviewed blind by one of the authors (David Rendle). Treatment responses were compared between horses that received 2 or 4 injections of 4 mg/kg LAIO at weekly intervals, and horses that received ORLO at 4 mg/kg PO SID for 4 weeks. Data were compared using a Mann–Whitney test with post hoc Dunn's test, chi‐squared test or Fisher's exact test.

**Results:**

Fifty‐six horses met the inclusion criteria: 29 received LAIO and 27 received ORLO. Treatment groups were comparable in terms of signalment and ESGD lesions. There was a difference in rate of healing when LAIO and ORLO treatment groups were compared at 28 days (LAIO‐97%; ORLO‐67%; *p* = .005; OR = 14(1.8–158)), but no difference between LAIO at 14 days and ORLO at 28 days (LAIO‐86%; ORLO‐67%; *p* = .12; OR = 3.1 (0.9–10)). Five localised and self‐limiting injection site reactions were identified in 3 horses out of 98 injections (5.1%).

**Main limitations:**

The study was limited by its retrospective nature, absence of randomisation and limited numbers.

**Conclusions:**

Four weeks of treatment with LAIO resulted in better rates of ESGD healing than 4 weeks of ORLO. Larger more robust studies of LAIO are warranted.

## Graphical abstract

A retrospective clinical study comparing rates of ESGD healing and improvement between oral omeprazole and a long‐acting injectable omeprazole preparation in 56 horses. Ninety‐seven per cent of horses treated with long‐acting injectable omeprazole healed within 28 days compared with 67% of horses treated with oral omeprazole. In conclusion, 4 weeks of treatment with long‐acting injectable omeprazole resulted in better rates of ESGD healing than 4 weeks of oral omeprazole.

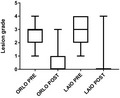

## 1 INTRODUCTION

Equine squamous gastric disease (ESGD) forms part of the equine gastric ulcer syndrome (EGUS) along with equine glandular gastric disease (EGGD). ESGD is a common condition in all types of horses across different athletic disciplines, with prevalence increasing in association with increased levels of exercise (Sykes, Hewetson, Hepburn, Luthersson, & Tamzali, 2015). Acid suppression is the mainstay of treatment for ESGD with proton pump inhibitors being the most effective means of achieving this aim (Sykes, Hewetson, et al., 2015). Omeprazole is the only proton pump inhibitor licensed for use in horses, and oral omeprazole paste (ORLO) is one of the most widely used medications in equine practice. Regardless of the formulation or dose used, 13%–27% of horses will fail to respond to 4 weeks of treatment with ORLO (Andrews et al., 1999; Doucet, Vrins, Dionne, Alva, & Ericsson, 2003; Lester, 2005; MacAllister et al., 1999; Sykes Sykes, & Hallowell, 2014, 2015). With the prevalence of ESGD being 80%–100% (Begg & O'Sullivan, 2003; Murray et al., 1997; Vatistas et al., 1999) in some populations, ORLO treatment failure is an important cause of morbidity and poor performance in equine practice.

Parenteral administration of omeprazole overcomes some of the factors that may limit clinical responses to oral omeprazole such as degradation in the stomach, reduced bioavailability with concurrent feeding and variable absorption from the gastrointestinal tract (Daurio et al., 1999; Murray, Nout, & Ward, 2001). The use of daily intravenous administration of omeprazole has been reported (Andrews et al., 2006) but the short half‐life and need for daily intravenous injections limits the practical application of this form of treatment in equine practice. Recently, the use of a long‐acting intramuscular omeprazole preparation (LAIO) that is administered weekly has been reported (Sykes, Kathawala, et al., 2017). This preparation was reported to have superior pharmacodynamics to ORLO, and the use of 2 doses of LAIO 7 days apart was associated with 100% healing in 22 Thoroughbred racehorses with ESGD (Sykes, Kathawala, et al., 2017). The following study evaluated LAIO in a different population of horses. The aim of the study was to determine whether LAIO was as effective in treating ESGD as ORLO. It was hypothesised that LAIO would be non‐inferior to ORLO for ESGD healing.

## 2 MATERIALS AND METHODS

### 2.1 Horses

Case records and gastroscopy images of horses presenting to Rainbow Equine Hospital for gastroscopy that were treated with ORLO or LAIO between May 2017 and May 2018 were retrieved and reviewed. Cases were excluded if clinical records or gastroscopy images were incomplete. Gastroscopy images were anonymised prior to review by one of the authors (David Rendle) who was blinded to the treatment allocation and stage of treatment. Treatments (LAIO or ORLO) were chosen by the attending clinician in discussion with the owner and with informed owner consent. ORLO is licensed in the United Kingdom for the treatment of ESGD and was used unless there was an indication to use LAIO. Indications for use of LAIO included an absence of improvement with previously administered ORLO for a reasonable time period, concurrent EGGD, an inability to fast the patient for an appropriate time period prior to administration of ORLO or difficulty administering oral paste preparations due to owner or patient factors. All owners were issued with a standard set of feeding and management instructions aimed at reducing risk factors for EGUS. Owners were advised to minimise exercise, maximise turn‐out, provide constant access to forage during the day, eliminate cereals from the diet and supplement the diet with oil at up to 1 ml/kg bwt per day, to provide additional calories in place of high starch feeds (unless the horse was overweight).

Horses that received LAIO were weighed on an electronic weigh scale and were injected with 4 mg/kg bwt IM of a 100 mg/ml omeprazole formulation^1^ weekly into the gluteal muscles. Gastroscopy was repeated at 2 weeks, after the initial 2 injections, and, if lesions had not resolved, gastroscopy was repeated again at 4 weeks after 2 further weekly injections had been administered. Horses that received ORLO were given 4 mg/kg of a licensed ORLO paste^2^^,^ 3 q24 hr PO for 4 weeks after which gastroscopy was repeated.

### 2.2 Gastroscopy

Gastroscopy was performed using a 3m flexible videoendoscope_._ 4 ^,^ 5 Gastroscopic examination included evaluation of the squamous mucosa including both the greater and lesser curvatures, as well as the glandular mucosa at the level of the margo plicatus and the pyloric antrum. A variable portion of the glandular body was visible due to incomplete emptying of gastric fluids despite fasting. Horses which did not have a complete set of gastroscopy images which showed the squamous mucosa of the greater and lesser curvatures were excluded. Lesions were graded using an accepted 0–4 scale (Sykes, Hewetson, et al., 2015).

### 2.3 Statistical analysis

Clinical data were recorded in Microsoft Excel™.^6^ Data for age and time between gastroscopic examinations were assessed for normality using a Shapiro–Wilk test and as they were not normally distributed, were evaluated using a Mann–Whitney test and post hoc Dunn's test. Gender, breed, horse use and presenting signs were compared using chi‐square test. Baseline data for gastric lesion scores were compared using a chi‐square test. Wilcoxon paired test was used to assess changes in lesion scores within groups over time, and either a chi‐square (if > 80% of the groups have a frequency of 5 or greater) or a Fisher's exact test (when < 80% of the groups have a frequency of 5 or greater) were used to evaluate healing, improvement and worsening of lesions between groups and associations with resolution of clinical signs.

Data are presented as median and inter‐quartile ranges (IQR) for continuous data when non‐normally distributed. Odds ratios (OR) and 95% confidence intervals (95% CI) are displayed for binomial data. Two commercially available statistical software packages were used.^7^^‐^ 8 Significance was determined when *p* < .05. Non‐inferiority statistics were performed to compare the two treatments with a significant difference between groups being 20% or more regarding lesion healing and improvement. Ninety per cent confidence intervals are displayed using Jeffrey's intervals and calculated using online statistical software.^9^ An a priori margin of 20% is commonly used when studies of this nature have not previously been published in the literature (Allen & Seaman, 2007) and was used for studying different doses of omeprazole in the horse (Sykes, Sykes, et al., 2015). A recent similar study evaluated a placebo versus misoprostol for the prevention of NSAID‐associated gastrointestinal injury in healthy volunteers, and the margin was similarly set at 17% (Lee et al., 2011).

### 2.4 Animal Ethics

This study underwent ethical approval from the University of Nottingham Clinical Ethical Review Panel. Informed consent from the owner was obtained for the use of LAIO and for the use of anonymised clinical data. A client information leaflet was provided to the owners of horses treated with LAIO https://www.beva.org.uk/Portals/0/Documents/ResourcesForVets/CILS/fInjectableOmeprazole.pdf?ver=2018-01-27-205616-937. Clients were advised to make contact immediately if any adverse events were suspected or noted.

## 3 RESULTS

### 3.1 Horses

Fifty‐six horses aged from 5 to 22 years met the inclusion criteria: 29 in the LAIO group and 27 in the ORLO group. Twenty‐five horses (21 in the LAIO group and 4 in the ORLO group) included in the current study had concurrent EGGD and were also included in a parallel study. There was no difference in age between the two treatment groups (LAIO–9 (IQR = 7–11) years and ORLO‐10 (IQR 8–14) years; *p* = .23). The distribution of mares to geldings was not different between groups (LAIO‐12 mares, 17 geldings and ORLO‐11 mares, 16 geldings *p* > .99; OR = 1.03 (0.36–3.0)). A wide range of breeds were represented with the most prevalent types being Thoroughbreds and Thoroughbred crosses (LAIO‐59%; ORLO‐48%), Warmbloods (LAIO‐24%; ORLO‐26%) or Cobs (LAIO‐7% ORLO‐ 22%), and there was no difference between treatment groups (*p* = .33). Horses were used for a variety of disciplines including general purpose riding (LAIO‐59%; ORLO‐30%), show‐jumping (LAIO‐21%; ORLO‐11%), dressage (LAIO‐7%; ORLO‐19%), pony club (LAIO‐0%; ORLO‐11%), hunting or eventing (LAIO‐3%; ORLO‐26%) and racing (LAIO‐10%; ORLO‐4%). There was a difference in discipline between the two treatment groups (*p* = .02). Six horses in the LAIO group had failed to respond to previous treatment with oral omeprazole immediately prior to initiating treatment with LAIO.

### 3.2 Presenting signs and improvement with treatment

The most common clinical complaints noted by the owner in both treatment groups were poor performance (overall–41%; LAIO–38% and ORLO–44%) and changes in behaviour (overall–32%; LAIO–28% and ORLO–37%). Other presenting signs included girthing pain (overall–25%; LAIO–21% and ORLO‐30%), signs of abdominal pain (overall–25%; LAIO‐24% and ORLO‐30%), weight loss or poor weight maintenance (overall–20%; LAIO‐21% and ORLO‐19%) and changes in appetite (overall–21%; LAIO–10% and ORLO‐33%). Fifty‐seven per cent of horses had two or more of the above clinical signs (LAIO‐41% and ORLO‐74%; *p* = .01 (OR = 0.25 [0.08–0.73])) with girthing pain and poor performance (overall–19%; LAIO–36% and ORLO‐10%; *p* = .17 (OR = 4.5 [0.8–26])) and poor performance and changes in behaviour (overall–34%; LAIO–27% and ORLO‐40%; *p* = .44 (OR = 2.4 [0.5–10])) being the two most common combinations of clinical signs.

Overall, there was an association between healing (*p* = .0001; OR = 18 (3.6–87)), but not simply improvement (*p* = .07; OR = 7.3 (0.97–92)) in lesion severity and the resolution of clinical signs in all treated horses. There was no association between healing (*p* = .18; OR = 5.3 (0.62–38)) or improvement (*p* > .99; OR = 0 (0–35)) of lesions and resolution of clinical signs at the first re‐examination (14 days (IQR = 14–16)) in the LAIO group. There was however an association between healing or improvement (*p* = .04; OR = ∞ (1.6‐∞)) and resolution of clinical signs by 28 days in the LAIO group. There was also an association between healing of lesions and resolution of clinical signs in the ORLO group at 28 days (*p* = .001; OR = ∞ (4.3‐∞)), but not with improvement of lesions (*p* = .12; OR = ∞(0.65‐∞)).

### 3.3 Gastroscopy

#### 3.3.1 Lesion scores

**Figure 1:**
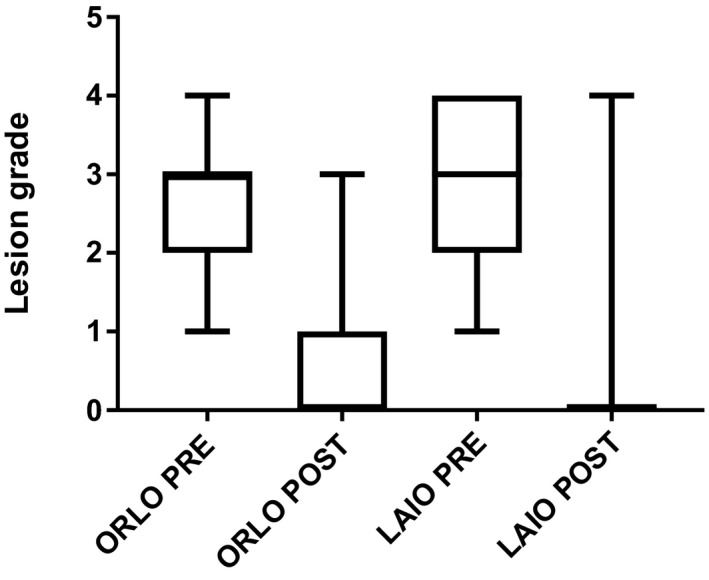
Equine squamous gastric disease lesion grade for both oral and long‐acting injectable omeprazole groups pre‐ and post‐treatment

The entire squamous mucosa was examined in all horses. There was no difference in lesion distribution between the two treatment groups (both greater and lesser curvatures (LAIO‐ 59%; ORLO −50%); lesser curvature (LAIO‐34%; ORLO‐27%); greater curvature (LAIO‐7%; ORLO‐23%; *p* = .24)). There was no difference in starting lesion score between the two groups (LAIO–3(IQR 2–4); ORLO–3(IQR 2–3); *p* = .06). Data for median (IQR) lesion scores pre‐ and post‐treatment are displayed in Figure 1. There was a reduction in lesion score in both treatment groups (LAIO—pre‐3(IQR 2–4) and post‐0 (IQR 0–0), *p* < .0001; and ORLO—pre‐3(IQR 2–3) and post‐0(IQR 0–1), *p* < .0001). There was no difference between treatment groups in the absolute final lesion grade (*p* = .06), but there was a greater reduction in the squamous lesion score in the LAIO group at 14 days (*p* = .02) and 28 days (*p* = .0001). There was a difference in rate of healing when LAIO and ORLO treatment groups were compared at 28 days (LAIO‐97%; ORLO‐67%; *p* = .005; OR = 14 (1.8–158)), but no difference between LAIO at 14 days and ORLO at 28 days (LAIO‐86%; ORLO‐67%; *p* = .06; OR = 4.2 (0.9–16)). There was no difference in rate of improvement when LAIO and ORLO treatment groups were compared at 28 days (LAIO‐100%; ORLO‐89%; *p* = .11; OR=∞(0.97‐∞)). LAIO at 28 days was found to be non‐inferior to ORLO at 28 days, for complete healing, but not improvement in lesion severity of squamous lesions (Table 1).

**Table 1:** Non‐inferiority analysis of treatment failures when the control (oral omeprazole) was compared with the novel treatment (long‐acting injectable omeprazole) for treatment of squamous gastric disease. The injectable omeprazole treatment failure for healing but not improvement could be shown to be non‐inferior to oral omeprazole.

	Failure rates		Difference in failure (%): Control treatment failure (omeprazole) minus novel treatment (injectable omeprazole) %	Upper 90% confidence interval
	Oral omeprazole at 28 days	Injectable omeprazole at 28 days		
Squamous healing	33% (9/27)	3% (1/29)	30%	40%
Squamous improvement	11% (3/27)	0% (0/29)	11%	19%

### 3.4 Adverse events

Localised swelling at 5 injection sites in 3 horses was identified. Ninety‐eight injections were administered giving a complication rate of 5.1%. No specific medical intervention was required with any injection site reaction. No adverse events were reported with oral treatment.

## 4 DISCUSSION

In the population studied, administration of four doses of LAIO given at weekly intervals was non‐inferior to 28 days of ORLO proving the initial hypothesis. No difference was identified between two doses at weekly intervals of LAIO and 4 weeks of ORLO. Use of LAIO was associated with a low complication rate. All reactions reported resolved without specific treatment.

Long‐acting injectable omeprazole has been demonstrated to suppress acid production more markedly, more consistently and for longer than observed in previous investigations using ORLO (Sykes, Kathawala, et al., 2017; Sykes, Underwood, McGowan, & Mills, 2015) so the difference between treatments seen in the current study was expected. In the current study, excluding 1 horse that was subjected to euthanasia as a result of lameness, all horses with ESGD healed after 4 weeks of LAIO treatment in line with the 100% healing rate reported in a previous study when horses with ESGD were treated with LAIO for 2 weeks (Sykes, Kathawala, et al., 2017). After two weeks of treatment in the current study, not all lesions had healed, but resolution in 86% of cases was comparable with the 100% (95% CI 89%–100%) reported by Sykes et al. (2017) given the small case numbers in both studies. Little work has been performed into why some horses fail to respond to treatment with proton pump inhibitors and in addition to failure of acid suppression, factors such as diet, exercise and changes in bacterial populations have been proposed as possible reasons for treatment failure in horses (Frank, Andrews, Elliott, & Lew, 2005; Jassim & Andrews, 2009; Lorenzo‐Figueras & Merritt, 2005; Luthersson, Nielsen, Harris, & Parkin, 2009; Vatistas et al., 1999). Factors other than inadequate acid suppression may have accounted for the absence of healing in some horses after 2 weeks of LAIO treatment in this study. However, the positive responses which result when the duration of treatment with proton pump inhibitors is increased and when LAIO is used in lesions that are refractory to ORLO suggest that inadequate acid suppression is likely to be the principal factor in these non‐responders.

Parenteral administration of omeprazole for the treatment of ESGD had been reported (Andrews et al., 2006; Jenkins et al., 1992; Sandin, Andrews, Nadeau, Doherty, & Nilsson, 1999) prior to the recent report of LAIO (Sykes, Kathawala, et al., 2017). The administration of soluble omeprazole preparations results in a significant increase in gastric pH; however, there is marked variability between individual horses and changes in pH are inconsistent (Andrews et al., 2006; Jenkins et al., 1992; Sandin et al., 1999). Intravenous omeprazole has a half‐life of approximately 30 min (Sandin et al., 1999; Sykes, Underwood, et al., 2015) and as a result the area under the plasma time curve, which is thought to be critical to clinical efficacy, is low. Further concerns with the use of soluble omeprazole are omeprazole's unstable nature when re‐constituted and its high pH which renders it potentially irritant (Jenkins et al., 1992).

The 67% rate of healing reported with ORLO in the current study is slightly lower than the 73% to 87% rates which have been reported previously after 28–35 days of ORLO (Andrews et al., 1999; Doucet et al., 2003; Lester, 2005; MacAllister et al., 1999; Sykes, Sykes, & Hallowell, 2014; Sykes, Sykes, et al., 2015). The reason for this slight difference is unclear but may be a result of the relatively low numbers in the current study, differences in the definition of healing or possibly a bias in the population in the current study which comprised horses that were treated at a referral hospital. Marked variation in acid suppression between horses in response to the same doses of ORLO have been reported regardless of the route of administration (Sykes, Kathawala, et al., 2017), and in some horses, ORLO may not stimulate sufficient acid suppression to permit healing of ESGD lesions (Sykes & Hallowell, 2019). When omeprazole is administered via an oral route, variation between horses may be compounded by differences in bioavailability which can vary up to 10‐fold (Sykes, Underwood, et al., 2015). Although initial reports indicated that oral omeprazole suppressed acid production for the 24‐hr period between doses (Daurio et al., 1999; Jenkins et al., 1992), more recent studies suggest that for at least half the 24‐hr treatment interval there may be insufficient acid suppression to promote healing of the squamous mucosa (Merritt, Sanchez, Burrow, Church, & Ludzia, 2003), particularly in horses that are fed a high forage diet (Sykes, 2016).

Variability in management and feeding are potentially confounding factors as the rates of healing identified with oral omeprazole in the current study are similar to those reported previously when horses were not fasted prior to the administration of omeprazole (Sykes et al., 2014; Sykes, Sykes, et al., 2015). Poor compliance with the advice given regarding management and feeding practices, would have the potential to impact negatively on treatment responses with ORLO. Forage feeding can markedly reduce the bioavailability of oral omeprazole (Sykes, Underwood, Greer, McGowan, & Mills, 2017) so failure to fast horses prior to treatment or to correctly co‐ordinate the timing with omeprazole administration could reduce the response to ORLO; however, this reflects the true situation in clinical practice. All owners were provided with a set of standard feeding and management recommendations and indicated that they had complied with these recommendations; however, it was not possible to verify that this was true. The use of more than one ORLO product might also be considered confounding; however, the two products used are bioequivalent (Norbrook Animal Health, unpublished data) so there is no reason to suspect a difference in clinical response.

The absence of prospective random allocation to a treatment group limits the study. Within the United Kingdom, it is not possible to use unlicensed medicines in such a manner legally so the only means by which the efficacy of the two omeprazole treatments could be compared was in a retrospective review of clinical cases in which unlicensed medicines have been administered in accordance with the legal framework on a case by case basis. While there was no difference in age, sex or breed distribution between the two groups, there were differences in numbers of horses performing different disciplines between groups. The differences between groups are probably an anomaly resulting from the low number of horses in each group; horses performing high‐ and low‐intensity exercise were equally represented in both groups so it seems unlikely this would have had a major confounding effect on the response to treatment. Bias in the allocation of cases may have influenced the results. As LAIO was perceived to be more effective based on previous reports (Sykes, Kathawala, et al., 2017), horses with more severe disease were more likely to receive LAIO. Six horses in the LAIO group had failed to show any response to ORLO and it is logical that these horses would be less likely to respond to treatment with a different omeprazole formulation. Conversely, it could be argued that premedication with ORLO might have resulted in partial improvement favouring further improvement and/or healing in horses in the LAIO group; however, horses which had shown a favourable response to ORLO continued on this treatment rather than having treatment changed to LAIO. Horses with concurrent EGGD were over‐represented in the LAIO group. Although there is no known reason why the concurrence of EGGD would affect ESGD healing, understanding of EGGD is poor and delayed gastric emptying has been proposed as a mechanism by which EGGD might be related to ESGD (Sykes, Bowen, Butcher, Green, & Hallowell, 2019). Responses to treatment for EGGD in the same population of horses are discussed in a parallel study (S. Gough, unpublished data 2019). Delayed gastric emptying was not observed in horses in the current study, and other investigations have failed to identify a relationship between ESGD and EGGD (Murray et al., 2001).

In conclusion, in a population of sports and leisure horses, 4 weeks of treatment with LAIO was a more effective treatment for ESGD than 4 weeks of ORLO and resulted in healing of ESGD lesions in all but one horse that was subjected to euthanasia as a result of severe lameness. When two weeks of LAIO and 4 weeks of ORLO were compared, the difference was not significant. Case numbers in both groups were low and the study was subject to a number of important limitations. Larger, blinded, randomised clinical trials of LAIO are warranted.

## CONFLICT OF INTERESTS

D. Rendle has previously received payment for consultancy services provided to BOVA UK and Luoda pharma who produce the LAIO and from Boehringer Ingelheim and Norbrook Animal Health who produce oral omeprazole.

## ETHICAL ANIMAL RESEARCH STATEMENT

Informed client consent was obtained from all owners to use an unlicensed product and to publish clinical data. The study was approved by The University of Nottingham Animal Welfare and Ethical Review Body.

## Data Availability

Data are available from the corresponding author on reasonable request.

## ENDNOTES

1 Injectable omeprazole. BOVA UK, 7 Gorst Rd, Park Royal, London, NW10 6LA.

2 Peptizole. Norbrook Animal Health, Norbrook Laboratories Limited, Carnbane Industrial Estate, Newry, Northern Ireland, BT35 6QQ.

3 Gastrogard. Boehringer Ingelheim, Ellesfield Ave, Bracknell, RG12 8YS.

4 Pentax B12303. Pentax House, Heron Drive, Langley, Slough, SL3 8PN, United Kingdom.

5 Aohua Vet 1,335. Aohua Endoscopy Ltd, Shengang Road, Songjiang District, Shanghai, 201,612, China.

6 Excel. Microsoft Corporation, Redmond, Washington, United States.

7 GraphPad 4.0. Graphpad Software, La Jolla, California, USA.

8 SPSS for Windows 15.0. SPSS Inc, Chicago, Illinois, USA.

9
://epitools.ausvet.com.au/content.php?page=CIProportion
